# Complete Genome Phasing of Family Quartet by Combination of Genetic, Physical and Population-Based Phasing Analysis

**DOI:** 10.1371/journal.pone.0064571

**Published:** 2013-05-31

**Authors:** Julien Lajugie, Rituparna Mukhopadhyay, Michael Schizas, Nathalie Lailler, Nicolas Fourel, Eric E. Bouhassira

**Affiliations:** Department of Medicine and Department of Cell Biology, Albert Einstein College of Medicine, Bronx, New York, United States of America; Seoul National University College of Medicine, Republic of Korea

## Abstract

Phased genome maps are important to understand genetic and epigenetic regulation and disease mechanisms, particularly parental imprinting defects. Phasing is also critical to assess the functional consequences of genetic variants, and to allow precise definition of haplotype blocks which is useful to understand gene-flow and genotype-phenotype association at the population level. Transmission phasing by analysis of a family quartet allows the phasing of 95% of all variants as the uniformly heterozygous positions cannot be phased. Here, we report a phasing method based on a combination of transmission analysis, physical phasing by pair-end sequencing of libraries of staggered sizes and population-based analysis. Sequencing of a healthy Caucasians quartet at 120x coverage and combination of physical and transmission phasing yielded the phased genotypes of about 99.8% of the SNPs, indels and structural variants present in the quartet, a phasing rate significantly higher than what can be achieved using any single phasing method. A false positive SNP error rate below 10*E-7 per genome and per base was obtained using a combination of filters. We provide a complete list of SNPs, indels and structural variants, an analysis of haplotype block sizes, and an analysis of the false positive and negative variant calling error rates. Improved genome phasing and family sequencing will increase the power of genome-wide sequencing as a clinical diagnosis tool and has myriad basic science applications.

## Introduction

Construction of completely phased genomes remains difficult to achieve. Statistical approaches based on the exploitation of the haplotype structure of human populations [1], provide relatively accurate local phasing information for common variants but do not work for SNPs distant more than 150–250 kb because the first error breaks down the phasing chain.

Both physical and genetic transmission methods can be used to formally determine the phasing of variants. Physical methods are based either on experimentally separating the two haploid genomes prior to sequencing, or on pair-end sequencing of library of DNA fragments. Phasing has been inferred by imaging single molecules after long-range PCR [2], by sequencing extremely diluted samples [3] and by sequencing males gametes [4]. Three genome-wide physical phasing methods have recently been reported: chromosome sorting [5], sequencing of diluted pools of large insert library ( [6] and sequencing of libraries prepared from solution of extremely diluted genomic DNA [7]. The major advantage of a physical approach is that it can be applied even when family members are not available; however the requirement for extreme dilution followed by re-amplification lead to high error rates and uneven coverage depth across the genome.

Genome-wide transmission analysis to infer phasing was pioneered by Roach et al. on a quartet [8] and generalized to larger families by the same group [9]. Dewey et al. have recently applied a similar method successfully [10]. A major advantage of transmission phasing is that in addition to producing accurately phased genomes, it yields much more precise genome sequences because it allows the detection of most errors through analysis of the compatibility of the genotypes with the law of Mendelian inheritance and with the patterns of paternal and maternal chromosomal inheritance. Error detection is most efficient if at least four members of a pedigree are sequenced.

Deciphering the phase of all variants after whole genome sequencing is important for a number of reasons. First, several genetic diseases are dependent on the parental origin of alleles. Phased genomes greatly facilitate the identification of the genetic variants responsible for such diseases. Additionally, SNPs and structural variants causing disease are not all located within a protein coding sequence, but can be within *cis*-acting regulatory sequence instead. Identifying the target genes of such variants is difficult. Phased genomes can help identify functionally significant variants in *cis*-acting regulatory sequences particularly if allele specific functional data such as RNA-Seq are also available. Phasing information helps determine whether double heterozygous mutations affecting the same transcription unit are in *cis* or *trans*. This is critical since in the former case only one of the two alleles is likely to be functionally deficient, while in the latter case, both alleles could be deficient depending on the nature of the effect. Finally, phased data is also invaluable to understand gene flow and characterize variant effect at the population level.

Using current algorithms published by Roach et al and Dewey et al., state of inheritance analysis in quartet allows the phasing of about 95% of all variants in Caucasians because variants that are uniformly heterozygous in the four family members cannot be phased in any of the family members. In more ethnically diverse families the proportion of variants that can be phased by transmission is lower because the proportion of heterozygous variants is higher. Moreover, transmission analysis does not allow complete phasing of the parents chromosomes: Rather, the parent chromosomes can only be phased within blocks defined by the location of the crossing-overs detected in the children (Figure S1).

Here, we report a genome phasing method based on combining transmission analysis with analysis of pair-end reads from fragment libraries of different sizes and with population-based analysis. Combination of these three methods allowed us to phase almost all variants including 99.89% of the heterozygous SNPs and 94.4% of the quadruple heterozygous. We provide a complete analysis of the number of SNPs, indels and structural variants in the quartet, a detailed characterization of the rates of false negative and false positive calls and an analysis of haplotype sizes. We also provide an analysis of genes homozygously affected by SNPs, indels or structural variants expected to severely disrupt protein sequences.

## Results

**Figure 1:**
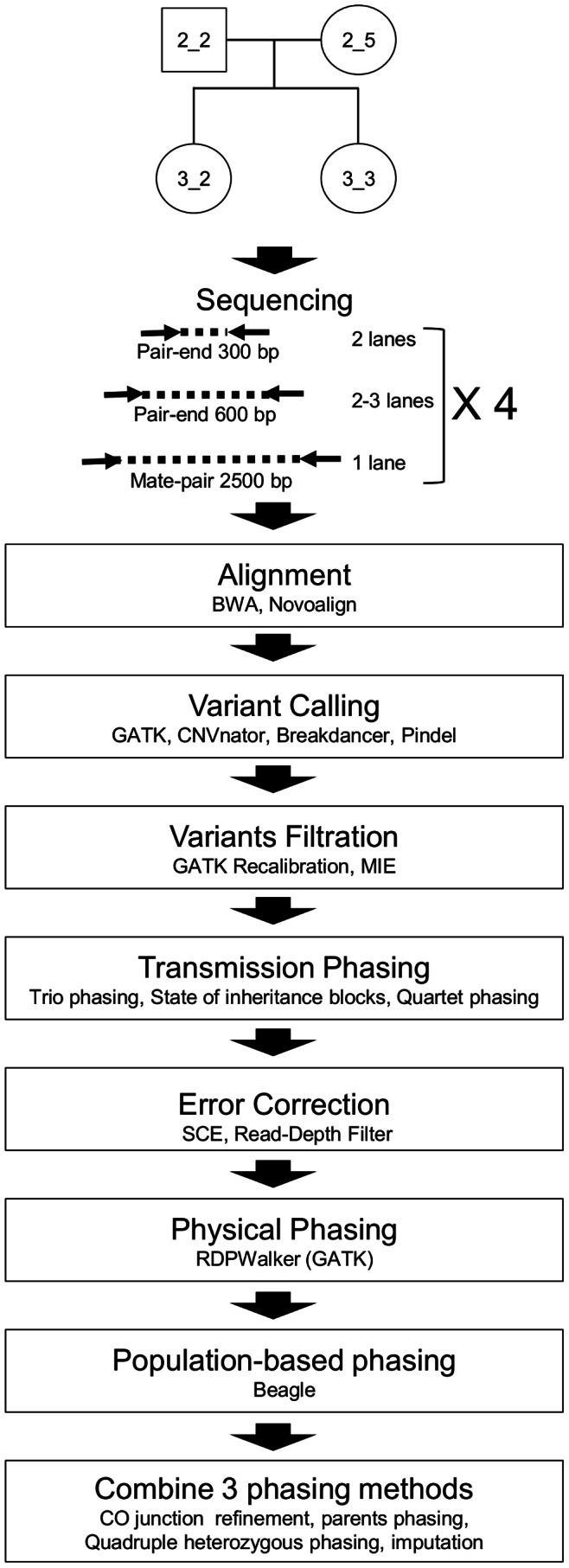
Sequencing strategy. Libraries of three different sizes were prepared from each member of the quartet from family FNY01 and sequenced on an Illumina HiSeq 2000. Reads were then aligned using BWA and Novoalign and variants were called and filtrated. Then, MIEs were detected. After that, variants were phased by transmission and errors were called. Phasing was then refined by physical and population-based approaches. Finally, phasing from all approaches was merged and recombination blocks and error analysis were refined. Positions called as MIE, SCE and uncalled or partially called positions were imputed by Beagle.

Informed consent from a healthy Caucasian family quartet (Family FNY-01, Figure S1) was obtained and DNA from peripheral white blood cells was extracted. Figure 1 describes the workflow used to generate the phased genomes. Pair-end libraries with average insert sizes of 300 bp, 600 bp and 2.5 kb (mate pair libraries) were prepared from genomic DNA from each member of the quartet. The pair-end libraries were sequenced on two to three lanes of an Illumina HiSeq 2000 while each mate-pair library was sequenced on only one lane. A total of 5.5*10^9^ 100-mer reads were obtained (Table S1) corresponding to coverage of more than 120 X for the quartet. The reads were then aligned to hg19 using BWA and Novoalign (for the mate-pair libraries) yielding 3.8*10^11^ uniquely aligned bases (4.0*10^9^ uniquely aligned reads). About 70% of the uniquely aligned bases were reported with a quality score above 35. The alignment rate averaged 90% for the pair-end libraries but was lower (about 48%) for the mate-pair libraries because of the complex structure of these libraries. SNPs and indels were called using the GATK software package [11]. Prior to any filtration, a total of 6,105,307 fully, partially, or uncalled variant positions were obtained (5,277,173 SNPs, 714,192 indels and 113,942 partially called variant) (Table S3).

### Phasing Analysis

**Figure 2:**
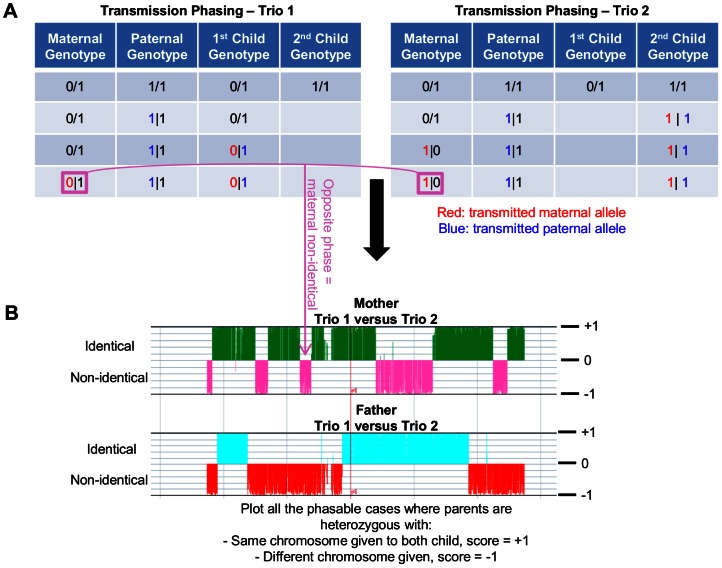
Phasing Strategy. (A) Trio Phasing: the quartet was divided into 2 trios and all phasable positions (i.e. positions with at least one homozygous member) were phased. The children genotypes were arbitrarily ordered as follow: Maternal Allele | Paternal Allele. The parent genotypes were arbitrarily ordered as follow: Transmitted | Not Transmitted Allele. For each phasable position, trios were phased in three steps: First, we marked all heterozygous genotypes as phased. Then, we use the heterozygous variant to phase a parent-child pair. Finally, we phased the second parent-child pair. (B) Blocks of Inheritance: X-Y scatter plots were created for each parent by assigning a value of +1 to each phased variant where the children inherited the same parental chromosome and a value of -1 to each phased variant where the children inherited different chromosomes. These graphs were used to define the preliminary blocks of inheritance (see Figure S2 for more details).

Transmission phasing was performed as follows. The quartet was first split in two mother-father-child trios and the variants were phased within each trio whenever possible, that is, when at least one member of the trio was homozygous for the reference or alternate allele (Figure 2A). Comparison of the two phased trios was then used to graph preliminary blocks of inheritance using GenPlay, a multi-purpose genome analyzer [12]. GenPlay provided a graphical visualization of the location of all the crossing-overs and some of the genetically detectable errors (GDE) present in the quartet (Figure 2B). The edges of these preliminary blocks of inheritance were formally defined using a segmentation algorithm available in GenPlay (island finder function). We then phased the triple heterozygous positions in each trio since such variants are not phasable without prior knowledge of the blocks of inheritance. This allowed us to refine the edges of the inheritance blocks (Figure S2). As shown in Table 1, about 91.30% of all heterozygous SNPs were phased with this approach. The remaining 154,264 unphased SNPs were heterozygous in all four family members which cannot be phased using a transmission method. 96.03% of all SNPs were phased by this approach.

**Table 1:** Phasing rate.

	Sample	Phased #	Phased %	Unphased #	Unphased %	Het. Phased #	Het. Phased %	Het. Unphased %
**Transmission Phasing**	**Quartet (avg)**	**3,727,607**	**96.03%**	**154,264**	**3.97%**	**1,620,312**	**91.30%**	**8.70%**
	FNY01_2_2					1,645,173	91.43%	8.57%
	FNY01_2_5					1,568,428	91.05%	8.95%
	FNY01_3_2					1,647,335	91.44%	8.56%
	FNY01_3_3					1,624,837	91.33%	8.67%
**Read-Backed Phasing**	**Quartet (avg**)	**2,766,908**	**71.28%**	**1,114,963**	**28.72%**	**671,303**	**37.61%**	**62.39%**
	FNY01_2_2	2,509,520	64.65%	1,372,351	35.35%	438,386	24.21%	75.79%
	FNY01_2_5	2,822,590	72.71%	1,059,281	27.29%	674,398	38.90%	61.10%
	FNY01_3_2	2,860,926	73.70%	1,020,945	26.30%	790,581	43.64%	56.36%
	FNY01_3_3	2,874,595	74.05%	1,007,276	25.95%	781,847	43.70%	56.30%
**Combined**	**Quartet (avg)**	**3,794,268**	**97.74%**	**87,603**	**2.26%**	**1,688,104**	**95.06%**	**4.94%**
	FNY01_2_2					1,711,834	95.13%	4.87%
	FNY01_2_5					1,635,089	94.91%	5.09%
	FNY01_3_2					1,713,996	95.14%	4.86%
	FNY01_3_3					1,691,498	95.08%	4.92%

Statistics are based on phasing of the stringently filtered list of 3, 900,000 SNPs (see text).

Combined: transmission and read-backed phasing methods combined.

Phased # and %: Number and percentage of SNPs phased.

Unphased # and %: Number and percentage of SNPs that could not be phased. All unphased positions are positions where the four members of the quartet are heterozygous.

Het. Phased # and %: Number and percentage of heterozygous SNPs that could be phased.

Het. Unphased %: Percentage of heterozygous SNPs that could not be phased.

Examination of the inheritance blocks revealed that there had been 85 crossovers in the maternal gametes and 54 in the paternal (Table S2). The average number of crossovers per chromosome was therefore 1.85 in the female gametes and 1.27 in the male gametes, confirming previous observations that the frequency of crossovers is higher in females [13]. The median length between two phased SNPs at the crossover junction was 2.6 Kb, the average 9.3Kb (Table S2).

#### Error rate analysis and number of SNPs in the quartet

Once inheritance state blocks were generated, we performed an analysis of transmission errors to calculate the number of true SNPs in the quartet and to compute error rates. Two major types of errors can be detected by transmission analysis: Mendelian Inheritance Errors (MIE) and State Consistency Errors (SCE). MIEs are sequencing errors that lead to a combination of genotypes within the quartet that are incompatible with Mendelian inheritance laws. For instance, an MIE will be detected when the parents are both homozygous and identical to the reference sequence and at least one of the two children is heterozygous for a SNP (Figure S3A). SCEs are errors that are compatible with Mendelian inheritance laws, but incompatible with the chromosome transmission map that is uncovered by phasing and crossover analysis. For instance, an SCE will be detected when a SNP is present on one of the two maternal chromosomes, absent in the father and it is absent in a child that inherited the maternal chromosome carrying the SNP (Figure S3B). SCEs cannot be distinguished from very closely spaced reciprocal crossovers, but such events are extremely unlikely. MIEs and SCEs are also undistinguishable from *de novo* mutations; however, since the rate of *de novo* mutation is extremely low (less than 100 per genome) we classified all SCEs and MIEs as sequencing errors. We did not attempt to detect *de novo* mutations.

Transmission error analysis can be used to estimate the total number of SNPs and indels in a quartet and the rate of false negative calls, if the proportion of errors that are genetically detectable is known. As discussed in supplementary methods (Text S1) and in accordance with Roach et al. [8], we estimate that transmission analysis detects about 75% of all errors.

Using the GRCh37 (hg19) human genome assembly and the GATK Unified Genotyper module on high-quality reads and without additional filters, we detected 5,163,231 fully-called genomic positions where a SNP was present in at least one of the four family members (Table 2 and Table S3). In addition, the genotypes could not be called in one or more of the four family members at 113,942 positions. The no-calls and the partially called positions were excluded from error rate calculations because we could not determine how many SNPs would have been at these positions and because many of these positions were imputed later in the analysis. Despite the imputation, it is likely that the exclusion of the no-calls and partially called positions slightly decreased the error rate. The error rates discussed below are therefore under-estimates.

**Table 2:** Error analysis, SNPs.

	SNP #	MIE #	MIE %	SCE #	SCE %	GDE-corrected	False positive	False negative	Ti/Tv	Ti/Tv Known	Ti/Tv Novel	DbSNP %
GATK Calls	5,163,231	153,552	2.91%	227,866	4.32%	4,781,813	2.66%	not applicable;	2.01	2.07	1.16	95.55%
Read Depth Filtration	4,958,825	139,757	2.76%	152,586	3.01%	4,666,482	2.07%	2.47%	2.03	2.08	1.15	95.91%
Recalibration (99 = PASS)	4,269,078	35,465	0.83%	25,935	0.36%	4,207,678	0.48%	13.64%	2.12	2.13	2.02	98.14%
PL Filtration >20	3,892,936	5,318	0.14%	5,408	0.07%	3,882,210	0.09%	23.17%	2.12	2.13	2.01	98.13%
Hemizygous Correction	3,892,936	4,713	0.12%	5,408	0.14%	3,882,815	0.09%	23.15%	2.12	2.13	2.01	98.13%

SNP # = Number of SNP called (total # of SNPs – # of partially called SNPs).

GDE corrected = Number of SNPs that are neither MIE nor SCE.

#of False positive: 1/3 of GDEs (MIEs+SCEs); FALSE positive % = 100*0.33*(MIE+SCE)/GDE-corrected SNP.

False negative %: 100*[4,781,813– [GDEs - corrected SNPs] (after filter)]/[GDEs - corrected SNPs] (after filter)]].

Ti/Tv known = transition to transversion ratio of SNPs found in dbSNP.

Ti/Tv novel = transition to transversion ratio of SNPs not found in dbSNP.

Transmission error analysis revealed 381,418 GDEs (153,552 (2.91%) MIEs and 227,866 (4.32%) SCEs). Since the number of GDEs represents 75% of all errors, it follows that there are at least 4,654,674 SNPs in the quartet (5,163,231 (number of called SNPs) minus 381,418 (the number of GDE) and minus 127,139 (the number of genetically undetectable errors, that is equal to 1/3 of the number of GDEs)). Subtracting the GDEs from the list of called SNPs provided a list of 4,781,813 GDE-corrected SNPs. This list of SNPs is referred to in the remaining of the text and in the figures as the 4.8 million SNP list.

From this list it is possible to compute a rate of false positive calls defined as positions where at least one of the genotype in a family member is wrong: Since there are 127,139 undetected errors in the 4.8 million SNP list, the rate of false positive calls is equal to 2.66% (100*127,139/4,781,813). To decrease the number of false positive calls we successively applied a series of filters. We first applied a read-depth filter created using GenPlay [12] and CNVnator [14]. This filter excluded the genomic regions that contained an abnormally high number of reads (at least twice as many reads) in all four individuals. This filter eliminated 115,331 SNPs including 89,075 GDEs. The efficiency of this filter (defined as the ratio of errors removed to the number of SNPs removed) was therefore 0.77. Application of this filter decreased the rate of false positive calls to 2.07% at the cost of an increase in the rate of false negative calls to 2.47%. The false negative percentage was defined as the ratio of the number of reads lost during the filtration step to the number of reads remaining after the filtration step (100*(4,781,813-4,666,482)/4,666,482).

We then applied the variant quality recalibration filter from the GATK which uses Gaussian mixture modeling of high-quality datasets to evaluate the probability that each call is real. This filter had an efficiency of 0.5 and decreased the false positive rate to 0.48% while increasing the false negative rate to 13.64%.

Finally, to decrease the number of false positive calls even further, we applied a filter based on the GATK-generated PL values. These values are measures of the likelihood that different genotypes, from the one reported, are correct. Using this filter we were able to decrease the false positive rate to 0.09% at the expense of a false negative rate of 23.17% (filter efficiency = 0.15). With this most stringent filter, 3,882,210 SNPs and approximately 3,575 undetected errors remained, leading to a false positive error rate per genome and per base equal to about 2.78*E-7. This list of SNPs is referred to in the remaining of the text and figures as the 3.9 million SNP list.

In summary, we detected about 4.8 million SNPs in the quartet with a false positive rate of around 2.66%. Filtration of these errors was possible and yielded a list of about 3.9 million SNPs that had very few false positives, but a high rate of false negatives. Both filtered and unfiltered lists of SNPs are useful for different applications. The number of SNPs that we found in this Caucasian quartet is slightly higher than what was previously reported: Roach et al. found 3,665,772 SNPs including 84,958 GDEs when comparing their quartet to the GRCh37 reference sequence, and Dewey et al. reported about 4,302,405 SNPs between their north European quartet and GRCh37. Since false negative error rates were not reported in these previous studies, and since Caucasian origin is a very loosely defined term, these numbers of SNPs cannot be directly compared.

When no filters were applied 95.55% of the SNPs were in DbSNP with a Ti/Tv ratio of 2.07 (Table 2). The 4.45% of novel SNPs had a Ti/Tv ratio of only 1.16 suggesting that a significant proportion of these novel SNPs are sequencing errors. When the read depth and GATK filters were applied the number of novel SNPs dropped to 2% and the Ti/TV ratio of the novel SNPs became greater than 2.0 which is equivalent to the Ti/Tv ratio of the known SNPs. This data suggests that there are at least 90,000 new SNPs in the quartet (2%).

We then estimated the false positive error rate using three other methods (Tables S4, S5, S6 and Text S1). First we analyzed the genomic regions that are identical by descent in the two children. These regions are of interest because the genomes of the children are sequenced three times and because all sporadic errors (i.e. errors that occurred in only one of the 8 sequenced haploid genome) are detectable in the children (but not in the parents). The false positive error rate calculated in these regions was 5.3*E-7 errors per base and per genome. We then analyzed the non-identical fraction of the quartet genome that contains the regions in which the children have inherited completely different chromosomes. These regions are of interest because they have been sequenced twice for each of the four members of the quartet and because they are the only regions in which all sporadic errors are detected in all family members by transmission analysis (Text S1). The average error rate in these regions for the entire quartet was equal to 1.32*E-06 errors per genome and per base. Finally, we analyzed the invariant fraction of the genome which contains the genomic positions where no SNPs have been reported. These regions can be used to estimate the fraction of errors that are sporadic errors. This analysis yielded an error rate of 2.92*E-07 per genome and per base. The discrepancy between the rates of false positive calls calculated with different approaches reflects the variability of the number of errors that are detectable by transmission in different fractions of the genome and the fact that most errors are not sporadic but are caused by local lack of coverage which often results in multiple errors at the same genomic position.

An important question was whether our false negative rate could be confirmed by using an independent technology. To answer this, we genotyped the father (individual FNY01_2_2 of Figure S1) of the quartet using Affymetrix human SNP arrays 6.0 and analyzed the concordance of the calls obtained by sequencing and by arrays (Text S1 and Table S7). This chip contains 900,000 SNPs that are all in DbSNP. The concordance between the two platforms was above 99.8% when the SNPs that were present in the most stringently filtered list (the 3.9 million SNP list) were examined. Since we had already shown that the sequencing approach has a very low false positive error rate, this suggested that the rate of error for the Affymetrix array in the order of 0.2% (assuming that most discrepancies were due to errors in the Affymetrix results). To validate our estimate of the number of false negative calls, we then examined the SNPs that were called by both platform, present in the unfiltered list (the 4.8 million SNPs list), but absent from the filtered list (3.9 million SNPs list). Of the 25,396 SNPs in this category, 96.65% were called with the same genotype by both the sequencing and the array technology. Since the error rate of the Affymetrix platform in our conditions was about 0.2%, this high concordance between the genotypes called by both platforms demonstrates that most of the SNPs that were filtered to eliminate errors were, in fact, present in individual FNY01_2_2, as suggested by the state of inheritance analysis. This important result strongly validates our estimate that there are about 4.66 million true SNPs in the quartet. As expected, concordance between the sequencing and array-generated calls was much lower at the positions flagged as MIEs or SCEs by the transmission error analysis (Table S7), again confirming the validity of the comparison between the two platforms.

### Hemizygous Correction

Heterozygous genomic deletions lead to homozygous calls by the GATK of variants that are in fact hemizygous. This creates MIEs that can be corrected once the structural variants (SV) are analyzed. Hemizygous corrections were performed after calling the structural variants (see below) and resulted in the correction of 605 MIEs in the highly filtered list of SNPs (Table S3).

### Physical Phasing

Physical phasing was performed using the read-backed-walker from the GATK (Figure S4). When the 3.9 million SNP list was analyzed, an average of 71.3±3.9% of all SNPs could be phased using information present in the pair-end sequencing data (Table 1). The N10 value of the fragments phased by read-backed phasing alone was 3.3 Kb and the N50 875 bp (Table S8). Comparison of the transmission and physical phasing methods revealed that the results were in agreement since there were less than 2,000 positions out of more than 3.9 million SNPs where the two methods diverged (divergence rate <0.059%) (Table S9).

Combining physical and transmission phasing methods allowed us to phase, on average, an additional 44% of the fully heterozygous SNPs in each quartet member. This increased the overall phasing rates to 98% without using any statistical inference method (Table 1). In addition, we were able to phase 18 recombination blocks out of 132 in the parent chromosomes (Table S10).

### Population-based Phasing

Allelic chromosomal segments in one or more individuals can be identical by descent or by ancestry. Genomic segments that are identical by descent are DNA fragment that can be traced directly to known ancestors such as parents and grand-parents. Genomic segments that are identical (or very similar) by ancestry are segments that contain variants in strong linkage disequilibrium because they were inherited from a common ancestor that lived an undefined number of generations ago. Genomic segments highly similar because of ancestry are called haplotypes and have been mapped using statistical inference in the major human populations. Haplotypes can be used to phase variants in the absence of any family information or can be used to complement transmission analysis. Haplotypes are also very useful in population genetic to track gene flow and understand the evolution of the human genome.

To phase the remaining unphased quadruple heterozygous variants and to impute the MIEs, the SCEs ad the uncalled positions, we then run Beagle [15], the genetic software analysis genome-wide. This software takes advantage of the HapMap and the 1,000 genome project to infer the genotype at positions where the sequencing data is incomplete. We first run Beagle on single individuals and then on trios. Of the 4,781,813 GDE-corrected SNPs about 94% (4,490,251) were in the 1,000 genome project and could, therefore, be analyzed using Beagle. As expected, comparison of quartet-based transmission phasing with Beagle-based phasing of individual quartet members revealed a good agreement for local phasing but very poor agreement for long-range phasing (Figure S5). We therefore focused most of the analysis on the results obtained when the two trios composing the quartet were analyzed using Beagle. In this mode, Beagle combines population analysis and transmission analysis and yield very accurate results. 5,731 of the 4,490,885 positions analyzed gave inconsistent results between the 2 trios. These positions were eliminated from the analysis since they represented Beagle phasing errors. Most of these errors were at the junction between haplotype blocks. After elimination of these errors, the concordance between the Beagle and the transmission plus physical phasing analyses was very high (99.9%). Integration of the Beagle imputed results with the transmission and physically phased results yielded phasing rate of 96.3% for the 4.8 million SNPs list and 99.8% for the 3.9 million SNPs list (Table 3). In addition Beagle was able to impute 121,593 GDEs or uncalled variants from the 4.8 million SNP list and 17,947 from the 3.9 million SNP list (Table 3).

**Table 3:** Heterozygous phasing and MIE, SCE and uncalled SNPs imputation with Beagle.

	GATK (filter: RDF)	GATK (Filter: RDF, PASS, PL>20 )
**PHASING**		
Total # of phased SNP^1^	4,666,482	3,882,210
SNP’s in HapMap	4,490,885	3,738,248
Number of unphased SNPs (quad. Het.)^2^	134,957	88,280
Number of unphased SNPs (quad. Het.) in HapMap	106,050	86,860
Phasing rate (transmission plus physical phasing)	93.1%	97.80%
Phased by Beagle^3^	101,687	83,409
Phasing rate (transmission plus physical phasing plus beagle)^4^	96.3%	99.8%
**IMPUTATION**		
Number of MIEs	139,757	5,847
Number of MIEs in HapMap	61,531	4,688
Number of MIEs imputed by Beagle	54,887	4,433
Number of SCEs	152,586	5,408
Number of SCEs in HapMap	39,775	3,941
Number SCE imputed by Beagle	34,934	3,661
Number of uncalled SNPs	104,500	14,416
Number of uncalled SNPs in HapMap	36,392	10,990
Number of uncalled SNP imputed by Beagle	31,772	9,853
Total # of Imputed positions	121,593	17,947

1: GDE-corrected SNP number minus number of variants filtered with RDF (See Table 2).

2: All unphased SNPs are quadruple heterozygous SNPs.

3: inconsistencies between trios were not included. Position that were MIE, SCE or uncalled after GATK analysis and that were in the HapMap are not included,

4: SNPs were first phased by transmission, then by physical phasing and then using Beagle.

Beagle analysis was then used to phase the recombination blocks in the parents that cannot be phased by transmission. Twenty-five junctions were phased with a high degree of confidence because the cross-over took place between regions carrying different haplotypes in the father. The remaining 29 junctions were phased with a lower degree of confidence because the two paternal chromosomes carried the same haplotypes in the region where the crossing-over occurred. In the case of the mother 26 junctions could be phased with a high degree of confidence. Once the blocks were phased a new tag, termed the AA tag, indicating the parental allele of origin for every SNPs, indels and SVs was added in the vcf file to the format field of almost all variants.

### Haplotype Structure in Family FNY01

As discussed above, haplotypes are important tools to track gene-flow and to understand genotype-phenotype relationships. When a phased genome is available, the size of each haplotype block, that is the size of the segment of DNA that are highly similar because of ancestry can be directly measured by comparing the phased chromosomes.

**Figure 3:**
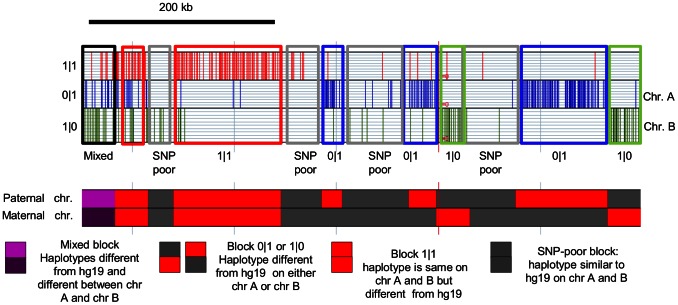
Haplotypes. Graphs illustrating haplotype structure in the quartet. A 550 Kb region of chromosome 5 is shown (chr 5∶97,464,122-98,064,122. Homozygous SNPs (red), heterozygous SNPs on chromosome A (blue) or heterozygous SNPs on chromosome B (green) were plotted and haplotypes were called using the island finder function of GenPlay. Regions in which islands were found only in the 1|1 track were named 1|1 blocks, regions in which islands were called only in either the 0|1 or 1|0 tracks were named 0|1 or 1|0 blocks. Regions where islands were found in either 1|1 and 0|1 or 1|1 and 1|0 or 0|1 and 1|0 or in all three tracks at the same time were named mixed-blocks. Regions in which no islands were found were named SNP-poor blocks.

To visualize the haplotype blocks, we plotted three parallel tracks in GenPlay the homozygous SNP (1|1 genotypes) and the SNPs that were present on either of the chromosomes (0|1 or 1|0 genotypes). As expected, this revealed a block structure that is equivalent to the haplotype structure (Figure 3) that can be defined by population studies. Haplotype block sizes were then estimated using the island finder algorithm in GenPlay.

As shown in Table 4, the average size of the genomic segment highly similar by ancestry was about 58,500 bp. About 36% of the genome of each individual was SNP-poor because it carried the same haplotype as the reference genome on both chromosomes. These regions had an average SNP density of 0.36 SNP/kb. About 18.35% of the genome of each individual consisted of regions where both chromosome haplotypes were different from one another and from the reference genome. These regions had a SNP density of 2.15/kb. The remaining 45.65% of the genome had a SNP density between 1.34 and 1.39 SNP/kb and consisted of regions in which one or two of the chromosomes carried one haplotype that is different from the haplotype of the reference genome. Haplotype definition on a phased genome is inherently more precise than the statistical analysis that is required for unphased genomes. As more phased genomes become available, studies of haplotypes defined without the use of statistical inference might help understand the evolutionary history of human populations and might help identify potential candidate SNPs in genome-wide association studies.

**Table 4:** Haplotype structure.

	0|1 Blocks	1|0 Blocks	1|1 Blocks	Mixed Blocks	SNP Poor Blocks	Genome Wide
Block #	8,027	8,800	10,723	10,186	13,930	51,666
SNP #	512,526	573,193	819,557	979,448	263,673	3,148,397
Total Length (bp)	379,895,027	427,998,919	629,719,719	577,878,545	1,133,640,956	3,149,133,166
Average Length (bp)	47,333	48,642	58,726	56,733	81,387	58,564
% Genome in Block	12.06%	13.59%	20.00%	18.35%	36.00%	100.00%
SNP Density (SNP/Kb)	1.39	1.38	1.34	2.15	0.36	1

0|1 Blocks = Blocks rich in heterozygous SNPs on the second allele.

1|0 Blocks = Blocks rich in heterozygous SNPs on the first allele.

1|1 Blocks = Blocks rich in homozygous SNPs.

Mixed Blocks = Blocks rich in heterozygous on either allele and rich in homozygous SNPs.

SNP Poor Blocks = Regions poor in SNPs.

### Indel Analysis

Calling indels with the GATK yielded 714,192 indels including 123,289 GDEs (Table 5 and Table S3) suggesting that there was about 590,903 true indels in the quartet. Using successively read-depth filtration, GATK variant recalibration, PL filtration and Hemizygous correction we were able to decrease the numbers of GDEs to 23,218 (5.18%). This yielded a list of 424,848 GDE-corrected indels with false positive and negative rates respectively equal to 1.80% and 39.09%. A list with a lower false negative rate, but a higher positive rate, could be obtained using less stringent filters (Table 5). After filtration, about 65.64% of the indels were found in the Mills and 1000 Genomes gold standard list.

**Table 5:** Error analysis, indels.

	Indel #	MIE #	MIE %	SCE #	SCE %	GDE-corrected	False positives	False negatives	Ins/Del	Ins/Del Known	Ins/Del Novel	Mills&1000G %
GATK Calls	714,192	31,858	4.46%	91,431	12.80%	590,903	6.88%	not applicable	0.94	0.86	1.05	56.31%
Read Depth Filtration	707,637	31,713	4.48%	89,079	12.50%	586,845	6.79%	0.69%	0.94	0.86	1.05	56.56%
Recalibration (99 = PASS)	668,869	29,068	4.35%	77,994	11.66%	561,807	6.28%	5.18%	0.93	0.86	1.06	58.66%
PL Filtration >20	448,066	3,016	0.67%	20,254	4.52%	424,796	1.80%	39.10%	0.89	0.84	1.01	65.64%
Hemizygous Correction	448,066	2,965	0.66%	20,253	4.52%	424,848	1.80%	39.09%	0.89	0.84	1.01	65.64%

Indel # = Number of indels called.

GDE corrected = Number of indels that are neither MIE nor SCE.

False positive = 1/3 of GDE (MIE #+SCE #).

False negative %: 100*[590,903– [GDEs - corrected] (after filter)]/[GDEs - corrected] (after filter)]].

Ins/Del = ratio of insertion to deletion.

Ins/Del Known = ratio of insertion to deletion in indels found in Mills and 1000G.

Ins/Del Novel = ratio of insertion to deletion in indels not found in Mills and 1000G.

### Histograms

Accuracy of variant calls can be estimated by plotting histograms of read-depth for the different genotype calls. To compare the SNP and indels calls accuracy, we plotted the read-depth frequency histograms for the 0/0; 1/1 and 0/1 calls (Figure S6). In the case of the SNP calls, the read-depth histograms were highly similar and symmetrical regardless of the genotype calls, suggesting that the GATK genotype caller did not introduce any major bias and that the calls are of high quality. Interestingly, the average read-depth for the position identified as MIE or SCE was lower, suggesting that some of these erroneous calls might be due to local low coverage. In the case of indels, the read-depth was higher for the homozygous 0|0 calls than for the heterozygous calls and for the 1|1 calls suggesting that a fraction of the read containing indels might not have been detected by the GATK and that the calls are of lower quality because they occur more often in region of low read depth. Therefore indel calling with the GATK remains less accurate than SNP calling despite the important improvement brought about by the realignment algorithm. Variant call accuracy can also be analysed by plotting histograms representing the ratio of reference to alternate allele found at every variant position. As shown in Figure S6, this analysis support the idea that the variant filtration improve the quality of the calls, confirmed that indel calling remain less accurate that SNP calling and suggests that the Bwa aligner and the GATK have minimal bias in favor of the reference sequence.

### Structural Variant Analysis

Once we had called the SNPs and indels using the GATK, we called the structural variants using a combination of Pindel [16], CNVnator [14], Breakdancer [17], GenPlay [12], and manual curation based on family inheritance analysis (Text S1). Since we did not perform a *de novo* assembly analysis, we could not detect insertion of novel sequences in the genome. MIE analysis could not be performed to assess error rate since compatibility with Mendelian laws was used as a criteria to call the SVs. State of inheritance analysis revealed that about 8.1% of the CNVs in the list were SCEs. Examination of these variants revealed that the vast majority were not false positives, but were located in complex genomic regions that contained multiple deletions in different quartet members that had led to genotype miscalling. These regions were labeled as SCEs and were not studied any further.

We detected 1,459 deletions that were heterozygous in at least one of the four members of the quartet (Table 6 and Table S11). As shown in Table 6, we found 12 deletions larger than 100 Kb, 102 between 100 Kb and 10 Kb, and 654 between 10 Kb and 1 Kb. About 20% of these SVs were novel since they were located in regions not previously reported to be prone to deletions.

**Table 6:** Structural variant numbers.

	Deletion #	Duplication #
Total	1,459	72
SVs >100 Kb	12	15
SVs between 10 Kb–100 Kb	102	25
SVs between 1 Kb–10 Kb	654	18
SVs between 100 bp–1 Kb	803	14
Known SVs	1,252	60
Novel SVs	319	17
SVs that do not affect a gene	879	21
SVs that affect a gene	580	51
SVs that affect at least one protein coding gene	546	38
SV that affect at least 1 exon in protein coding gene	125	38

We also detected 72 duplications (Table 6 and Table S12) including a 3.5 Mb duplication at the tip of chromosome 15 that contains about 32 ENSEMBL protein coding genes and a 1.03 Mb duplication at the end of chromosome 14 that contains 4 ENSEMBL protein coding genes. The number of insertions was much smaller than the number of deletions because we only looked for duplications. The true number of insertions in the four genomes studied is likely higher. Further analysis will be necessary to confirm this point.

We also called 3,718 regions as deletions or duplications that were homozygous in the four members of the quartet (Table S13). Further analysis revealed that a majority of these regions are not true genetic variants, but are caused by problems associated with the assembly of the genome, or by technical sequencing issues. However, some of these regions are likely to be true variants that are homozygous in all members of the quartet because the reference sequence represents a rare allele.

### Variant Effect

Table 7 describes the number of heterozygous and homozygous variants in each individual of the quartet. In the case of the stringently filtered 3.9 million SNP list, there was an average of 1,119,861±6,734 homozygous and 1,776,200±31,836 heterozygous SNPs between the reference genome and each of the individuals of the quartet. Comparison of the two parents in the quartet revealed the presence of 328,222 homozygous and 2,313,081 heterozygous SNPs between these two unrelated diploid genomes.

**Table 7:** Variant Genotypes.

		FNY01_2_2	FNY01_2_5	FNY01_3_2	FNY01_3_3	Quartet (Avg. ± Stdv)	2_2 Relative to 2_5
SNPs	Homozygous	1,120,406	1,129,224	1,110,198	1,119,615	1,119,861±6,734	328,222
	Heterozygous	1,799,927	1,723,224	1,802,157	1,779,490	1,776,200±31,836	2,313,081
Deletions (Indel)	Homozygous	59,068	58,980	58,046	58,329	58,606±431	19,001
	Heterozygous	109,928	106,840	114,241	112,672	110,920±2,816	131,983
Deletions (SV)	Homozygous	445	428	499	549	480±47	151
	Heterozygous	926	920	960	928	934±15	1,183
Insertions (Indels)	Homozygous	63,670	63,121	61,885	62,489	62,791±670	16,607
	Heterozygous	93,239	90,284	97,666	96,504	94,423±2,889	107,849
Insertions (SV)	Homozygous	15	18	14	11	15±3	7
	Heterozygous	36	32	38	39	36±3	60

2_2 Relative to 2_5 = Number of SNPs found in FNY01_2_2 when using FNY01_2_5 as reference.

Analysis using the SNPeffect [18] and SNPNexus [19] software revealed that, on average, each family member had 24,487 SNPs predicted to have either a high-impact (i.e. stop codon or splicing variant likely to strongly perturb expression levels) or a moderate impact (non-synonymous substitution) on a Refseq-defined protein-coding gene (Table 8) by the SNPeffect algorithm. Definition of high, moderate and low impact variants is described in Cingolani et al [18]. On average, there were 114.5±2.1 genes in each quartet member that were affected by a homozygous or double heterozygous high impact SNPs, likely to severely affect expression.

**Table 8:** Variant effects.

			FNY01_ 2_2	FNY01_2_5	FNY01_3_2	FNY01_3_3	Quartet average
SNPs	High impact	All genotypes	641	601	627	633	625.5±15.0
	Moderate impact	All genotypes	23,948	23,325	24,082	24,091	23861.5±314.9
	Modifier	All genotypes	31,474	31,193	31,852	32,022	31635.25±323.23
	High impact (homozygous)	Homozygotes	113	113	110	116	113±2.1
		Double-heterozygotes	3	1	1	1	1.5±0.9
		Double heterozygotes in trans	2	0	0	0	0.5±0.9
		Double heterozygotes in cis	1	1	1	1	1±0.0
		Phasing undetermined	0	0	0	0	0
	High impact+Moderate	Homozygotes	2266	2222	2231	2261	2245±18.9
		Double-heterozygotes	388	365	440	424	404.25±29.5
		Double heterozygotes in trans	107	88	121	113	107.25±12.2
		Double heterozygotes in cis	281	277	319	311	297±18.3
		Phasing undetermined	55	64	62	51	58±5.2
		Percent in trans	27.58%	24.11%	27.50%	26.65%	26.53%
		Percent in cis	72.42%	75.89%	72.50%	73.35%	73.47%
Indels	High impact	All genotypes	444	417	482	422	441.25±25.6
	Moderate impact	All genotypes	288	304	338	319	312.25±18.5
		Double (moderate/high impact)	38	38	40	37	38.25±1.1
	High Impact	Homozygotes	97	96	99	95	96.75±1.5
		Double-heterozygotes	1	2	1	1	1.25±0.4
		Double heterozygotes in trans	0	0	0	1	0.25±0.4
		Double heterozygotes in cis	0	1	0	0	0.25±0.4
		Phasing undetermined	1	1	1	1	1
		Percent in trans	0.00%	0.00%	0.00%	100.00%	50% ±0.4
		Percent in cis	0.00%	50.00%	0.00%	0.00%	50% ±0.2

High-impact variants were defined as variants that (1) formed or deleted a stop codon, (2) that altered a splice donor or acceptor site, (3) that caused a frameshift, or (4) that deleted one or more exon. Moderate-impact variants were defined as variants that altered a single amino-acid (non-synonymous substitution, codon insertion or deletion) or that deleted at least part of an exon.

A question that can only be answered when phasing information is available is whether double-heterozygous mutations affecting the same gene are located in *cis* or in trans. Out of an average of 404.25 double heterozygous high or moderate-impact mutations in each individual, 107.25 (27%) were in trans and 297 in *cis* (73%). The disproportionally high number of variants that were located in *cis* is caused by the haplotype structure of the human genome. This data demonstrate the value of phasing information for the evaluation of SNP effects.

### Indel Effect

Although the number of indels is only about 6% of the number of SNPs, an average of 96.75±1.5 genes or isoforms was affected by indels classified as high-impact (Table 8). This disproportionate impact of indels is due to the fact that small deletions or insertions tend to cause high impact mutations such as frameshift. Since indel calling is less accurate than SNP calling, some of the high impact mutations caused by indels might in fact be sequencing artifacts.

### Structural Variant Effects

About 10% (144/1571) of the deletions affected at least one protein coding genes and about 8% (107/1571) affected at least one exon. There were between 5 and 9 homozygous deletions that affected protein coding genes in each quartet member.

Adding together the effects of the three variants type analyzed, there were about 200 genes homozygously affected by a high-impact variant in each member of this quartet of healthy individuals. We then analyzed the variant effect on each splicing isoforms. Computation of the number of genes that had all their isoforms affected by a high impact variant yielded a list of 41 genes (Table S14) because all of the other high-impact variants affected only a fraction of the isoforms produced by a transcription unit. Of these 41 genes, eleven were homozygously affected in all member of the quartet and were likely mis-annotated in the reference sequence, twelve were olfactory receptors, single exon genes of unknown function or genes suspected of having an error in their gene model, and four were well-known polymorphic genes (RHD and HLA loci). The 14 remaining genes that contain homozygous high impact mutations had no or very weak disease association in the OMIM and other databases and most had no known functions.

## Discussion

We report here a method combining both genetic, physical and population-based approaches to phase the genomes of a quartet. The four haploid genome sequences that we have generated are among the most accurate published so far and false negative rates are reported for the first time. When transmission phasing was used alone, the phasing rate for heterozygous SNPs was about 91.5%. When genetic, physical and population-based methods were combined, the phasing rate reached 99.8%: In each individual, less than 4,800 SNPs were unphased out of average of about 1,800,000 million heterozygous SNPs.

Haplotype blocks were defined using an island calling algorithm to quantify the variation in SNP density along the chromosomes. When more phased genomes become available, such data should help reconstruct the evolutionary history of each haplotype block and help interpret association studies.

Using transmission error analysis, we were able to demonstrate that there are about 4.66 million true SNPs in the analyzed quartet. We provide a list of 3.9 million SNPs with virtually no false positive calls, but a large number of false negative calls (23%), and a list of 4.8 million SNPs with a higher false positive error rate but very few false negatives. Higher coverage or better sequencing technology would help decrease the false negative rate without increasing the false positive rate. Indel calling was significantly less accurate than SNP calling.

Transmission error analysis was very useful to call and genotype the structural variants. We generated a list of more than 1,450 genotyped structural variants. Availability of the quartet sequence was critical because it provided internal read-depth control.

Availability of phasing information allowed us to show that about 73% of all double heterozygous moderate or high impact SNPs that occurred within a transcription unit were compound heterozygous and therefore carried a wild type allele. Although all four members of the quartet are healthy individuals, we detected almost 200 high-impact variants within protein-coding genes in each member of the quartet.

Is family sequencing worth the expense? Quartet sequencing allowed us to achieve a very high rate of phasing and to define our false positive and false negative error rates. Our analysis required the use of three phasing methods and the construction of libraries of different sizes. Family information is essential to eliminate errors and calculate error rates. Generally read-back phasing was relatively inefficient, at least with the library sizes and the relative coverage for each library that we obtained. Higher read-backed-phasing efficiency might be obtained by adjusting the library sizes and the number of libraries sequenced. Sequencing a quartet and omitting the read-backed phasing step would eliminate the need to generate multiple libraries and would yield similar error rate but would slightly decrease phasing accuracy since SNPs that are not in the HapMap (about 6% of the detected SNPs) are not phasable by this approach.

It is difficult to estimate the cost of the production of a genome with similar precision by sequencing a single individual. Quartet sequencing at 120x coverage is roughly equivalent to sequencing two genomes at 60x coverage since the children genomes are contained within the parent genomes. In addition, the precision of the analysis is increased firstly because the GATK variant calling precision is higher when a quartet is sequenced since the variants are called as a group, and secondly because the availability of four genomes allows the generation of a highly efficient read-depth filter. Finally, at equivalent coverage, the number of undetectable errors would be about 4 times higher since transmission analysis allows the detection of about 75% of all errors. Phasing accuracy would also be much lower since as shown in Figure S5, population-based phasing without the help of family information yields accurate local phasing (up to 100–150 kb) but no useful long-range phasing information.

In summary, when family members are available, quartet sequencing combined with population-based phasing is a powerful, cost-effective method to obtain genome sequences with low error rates and with precise phasing. Read-backed phasing allows a refinement of the phasing but requires additional library construction and additional analysis. Production of a genome sequence with equivalent precision from a single individual would cost much more than the cost of quartet sequencing.

## Methods

### Sample Acquisition

Genomic DNA was prepared from 10 ml of whole blood using Wizard Genomic DNA Purification Kit (Promega), quantified using NanoDrop and PicoGreen method (Quant-iT PicoGreen dsDNA Assay Kit *2000 assays, Invitrogen).

### Pair-end Library Preparation

Genomic DNA was sheared to 300 bp and 600 bp size using Covaris, purified, and checked on bio-analyzer for size distribution. Each sample was end-repaired (End-It kit, Epicenter Biotechnologies), an A-overhang added (Klenow 3′ to 5′ exo minus, NEB), and ligated to illumine pair-end adapter sequences (Illumina). The ligated libraries were size selected (300±50 bp, and 600±50 bp), PCR amplified by 10 cycles, and purified with SPRI beads (Agencourt AMPure XP; Fisher Scientific). Libraries were quantified on a 1000 DNA Chip by 2100 bio-analyzer (Agilent Technologies, Inc.) and sequenced on Illumina HiSeq 2000.

### Mate Pair Library Preparation

Genomic DNA was sheared to 2.5 to 3 kb in size using Covaris, purified, and checked on bio-analyzer for size distribution. The mate pair libraries were generated as indicated in the Illumina protocol (Illumina, mate pair library prep kit). Briefly, Following DNA fragmentation, 2.5 kb fragments were end-repaired with biotin labeled dNTPs. The DNA fragments were circularized, and non-circularized DNA was removed by digestion. The Circular DNA was then fragmented to a size of about 300bp and biotin labels of the fragments (corresponding to the ends of the original DNA ligated together) were affinity purified. Purified fragments were end-repaired and ligated to Illumina Paired-End sequencing adapters.

### Affymetrix SNP Array

Genomic DNA from one of the family members was hybridized to genome-wide human SNP array 6.0 (Affymetrix). Analysis was performed using Genotyping Console software (Affymetrix). The software uses birdseed algorithm and hg19 annotation files to generate the final SNP list.

### Alignment to the Reference Genome

Alignments were performed using BWA and Novoalign. Default parameters were used for both aligners. BWA was used for all alignments except for the mate pair libraries which were aligned with Novoalign. All alignments were performed against GRC37 (hg19). Results of the alignments are described in Table S1. Quality scores are provided in Figure S7.

### SNP and Indel Calling

SNP and indel calling was performed using the GATK software suite (version 1&2). Parameters were generally set according to the suggested best practice. Read quality (Q) was set to 35 during the SNP calling step. Once the variants were called they were sequentially filtered as follows:

### Read-depth Filter

After alignment, a number of regions had abnormally high read-depth in all members of the quartet. These regions are mostly segmental duplications that were not incorporated in the reference genome. Variants cannot be genotyped in these regions because the aligned sequence represents the consensus sequence of several repeats that are not present in the reference genome. Attempt to genotype them yielded regions with high SCE density. To filter these error prone regions with high read-depth, we generated a filter that eliminates all the genomic regions that have higher than normal read-depth in all quartet members. (More refined analysis was performed subsequently to identify CNVs). The filter was created using CNVnator and the Island finder algorithm of GenPlay. About 18.8 * 10E6 bases, that is 0.66% of the genome (assuming a genome size of 2.86*10E9 non N bases) is covered by this filter.

### Variant Recalibration

Variant recalibration was performed following the best practice suggested by GATK.

### PL Filtration

Rate of MIEs and SCEs was used as a guide to maximize specificity defined as the ratio of MIE to non-MIE variants eliminated. All regions that were uncalled in at least one family member were filtered out at this step (some of these regions were reanalyzed and restored to the VCF files later in the analysis). The most efficient filters were found to be filters that eliminated calls in which at least one family member had a PL value below a certain value. Result for PL >10, 20 or 50 are summarized in Table S3. The PL >20 filtered data were selected for most of the subsequent analysis.

### Hemizygous Error Correction

Hemizygosity creates MIEs that can be corrected once the deletions causing the hemizygosity are correctly genotyped. 605 MIEs caused by hemizygosity in some of the individuals were corrected.

### Variant Effects

Variant effects were analyzed using a combination of SNPeffect [18]and SNPNexus [19]. ENSEMBL and RefSeq gene annotations were used.

### Detection of Structural Variants Greater than 100 bp

Structural variants were detected using Pindel [16], CNVnator [14] and Breakdancer [17] on a group of BAM files containing all the reads from each individual separately. In order to improve the sensitivity of the result, we also called the SVs on an additional group of files that contained all the reads from all the individuals together. In the case of Pindel, the mate pair libraries were not included in any of the groups because the software could not handle these files. Default parameters were used for all the programs**.** SVs were considered true positives if they were found by at least two programs in any of the group of files searched. Variants were considered true in the case which were found by two of the programs if they overlapped, regardless of the length. Overlap detection was performed using GenPlay and the Galaxy server. When variants overlapped they were given the sizes of the Pindel variants when available, or when Pindel had not detected the variants, the larger of the BreakDancer or CNVNator were used. Variants were extensively manually curated at the end of this analysis using read depth information and MIE information to eliminate incorrectly called or genotyped variants.

### Data Access

The raw sequence files, the 2 vcf files and the bed file with the family inheritance state blocks are available at NCBI BioProject database (http://www.ncbi.nlm.nih.gov/bioproject/191008) with Accession number: PRJNA191008 and ID: 191008 or at http://genplay.einstein.yu.edu/FNY01/(see file Text S1).

### Software Developed for this Project

Many of the computations necessary for this project were performed using GenPlay which is available at http://genplay.einstein.yu.edu/Specific code was written for error correction and phasing. The code can be found at https://genplay.einstein.yu.edu/svn/FamilyQuartet. Please inquire if you need help using the code.

The functions designed to facilitate phasing and error correction will eventually be incorporated within GenPlay.

## Supporting Information

Figure S1 **Parents are only partially phased by genetic phasing.** Phasing by transmission in a quartet allows complete phasing of the children except for positions that are quadruple heterozygous. In the case of the parents, the variants within blocks bounded by the crossovers in the children are phased but the blocks are not phased relative to each other, therefore, the parent chromosomes cannot be completely reconstituted. Physical method can be used to phase some of the quadruple heterozygous region as well as some of the crossover-defined blocks in the parents. The quartet represented illustrates the relationship of the four member of the healthy Caucasian quartet family FNY01. FNY01_2_2 = Father, FNY01_2_5 = Mother, FNY01_3_2 and FNY01_3_3 = 2 daughters.
(TIF)

Figure S2 **Triple heterozygous phasing.** The preliminary inheritance blocks were used to phase the positions in each of the two trios that could not be phased without prior knowledge of the inheritance blocks. After this final phasing step, the edges of the preliminary blocks were refined.
(TIF)

Figure S3 **Error analysis.** (A) Mendelian Inheritance Error: quartet genotype is incompatible with Mendelian laws. (B) State Consistency Error: quartet genotype is compatible with Mendelian laws but pattern of chromosomal inheritance is not.
(TIF)

Figure S4 **Physical phasing.** Heterozygous SNPs found on both ends of overlapping pair-end or mate-pair library fragments can be phased. Read-backed phasing was performed using the GATK package.
(TIF)

Figure S5 **Comparison of HapMap-based and quartet-based phasing.** Chromosome 1 of individual FNY01_2_2 was phased using Beagle using the hapmap 3.0 marker file and default parameters and the results were compared with our quartet based phasing. A value of one was assigned to concordant phasing between both methods and a value of negative one for the discordant phasing calls. The switch accuracy defined as the proportion of heterozygous positions misassigned relative to the previous heterozygous position [20] was equal to 3.5%. The first 5 million bases of chromosome 1 are shown.
(TIF)

Figure S6 **Histograms. (**A): Read-depth frequency histograms for 0|0 (REF,REF), 1|1 (ALT, ALT) and heterozygous calls in individual FNY01_2_2 are plotted for SNPs and indels. The 4,8 million SNP list was used after application of the read depth filter. The histograms are almost perfectly super-imposable, suggesting that the alternate (ALT) calls do not occurs preferentially in regions of low read depth. Interestingly the allele depth for the MIEs and SCEs is lower suggesting that the SNP calling errors occur more frequently in regions of low coverage. Hence, higher coverage could decrease the error rate even further. In the case of the indels, the read-depth is in average higher for the reference (REF) calls than for the ALT calls. This confirms that indel calling by the GATK is less precise than SNP calling. (B): Allele-depth frequency histograms. Log ratios of allele-depth are plotted. ALT calls #/REF calls # are plotted in the case of 0|0 and heterozygous genotypes calls. REF calls #/ALT calls # are plotted in the cases of the 1|1 calls. Histograms were generated using either the 4.8 million list of SNPs after application of the read depth filter only, or on the 3.9 million SNP list (RDF, PASS and PL>20 filters). Table on the right summarize average allele-depth ratios. In the case of the SNPs, average allele-depth ratios are very small for the 0|0 and 1|1 calls and close to 1 in the case of the heterozygous calls. As expected, application of more stringent filters yields smaller allele depth ratio in the case of the homozygous calls, and a ratio that is closer to 1 (from 0.93 to 0.98) in the case of the heterozygous calls. We conclude that, as expected, filtering erroneous calls increase the allelic balance quality of the calls. Indel calling is of lower quality than SNP calling. These results also suggest that alignment with BWA followed by variant calling with the GATK yields results with minimal bias in favor of the reference allele.
(TIF)

Figure S7 **Quality score.** Average quality scores and average quality scores greater than 35 for each individual. Average of the results for three libraries (of three different sizes), sequenced once or twice each, and is shown for each individual. X-axis: quality score. Y-axis: number of reads.
(TIF)

Table S1 **Alignment results.**
(XLSX)

Table S2 **Crossovers and Blocks.**
(XLSX)

Table S3 **Variant filtration.** GATK calls were filtered to decrease the rate of false positives (see Table 1 in main text). Efficiency of the filtration was evaluated by monitoring the numbers of GDEs. Filters were applied sequentially on the green lines (i.e. the raw variant calls were successively filtered using filters 2, 3, 4 and 5). Red lines: filters 3b, 4b and 4c are provided for information but were not used in the filtration chains.
(XLSX)

Table S4 **Error Rate in the Identical Blocks.**
(XLSX)

Table S5 **Error Rate in the Non-Identical Blocks.**
(XLSX)

Table S6 **Error Rate in the Invariant Positions.**
(XLSX)

Table S7 **Affymetrix Validation.** To validate the GATK calls and the error rate estimations we genotyped one of the individual of the quartet using the Affymetrix platform and calculated the discordance rate between the two platforms with or without filtration of the SNPs. The first row summarizes the results without filters: Of the 494,844 SNPs called by both platforms 493,014 were concordant and 1830 discordant (discordance rate = 0.37%). When the filters that reduce the false positive rate to 0.09% were applied (second row) the discordance rate drops even further to 0.21%.This is the expected result since the filters eliminates false positive calls. In the third row the SNPs that were filtered in the second row are analyzed. In that population of SNPs the discordance rate increased a little bit to 3.1% also as expected since the filtered SNPs should be enriched in erroneous calls. Importantly, the concordance rate in this list is above 96% suggesting that the vast majority of the filtered SNPs are false negative as predicted by the state of inheritance analysis. This result strongly validates our estimated of the rate of false negative and therefore of the total number of SNPs in the quartet. In the three last rows we analyzed the discordance rate for the SNPs that were flagged by the state of inheritance analysis as GDE. As expected the discordance rate is much higher (above 30%). The discordance rate is lower than would expected than by chance alone because calls were flagged as GDEs when an error was present in any one of the four quartet members. Therefore some of the calls flagged as MIE and SCE for the quartet might in fact be accurate in the particular individual (2_2) analyzed with the Affymetrix platform.
(XLSX)

Table S8 **Read-Backed Phasing N10 and N50.**
(XLSX)

Table S9 **Genetic and Physical Phasing Comparison.**
(XLSX)

Table S10 **Parent Block Phasing.**
(XLSX)

Table S11 **List of deletions larger than 100 bp and genotypes in the four quartet members.**
(XLSX)

Table S12 **List of duplications larger than 100 bp and genotype in the four quartet members.**
(XLSX)

Table S13 **List of regions with abnormal read-depth that were genotyped as homozygous in the four quartet members.** In addition to the deletions and duplications summarized in Tables S9 and S10, we also detected 3,717 regions with abnormal read-depth. The vast majority of these regions are not true structural variants but represent regions that have abnormal coverage for a variety of reason including gaps or misassemblies in the reference genome sequence, presence of low complexity repeats that are not well resolved by short-read sequencing or unknown segmental duplications. Some of these regions might also represent true SVs that were homozygous in all members of the quartet, either by chance or because the reference sequence represent a very rare allele. About 7.6% of all ENSEMBL genes were in regions that had abnormal read-depth and were detected by at least one SV detection software in all members of the quartet. Included in this list are a number of genomic regions (mostly near the centromere) that were not uniformly high or low read-depth but that were too complex to analyze for deletions and insertions because SV detection software gave contradictory results. These complex regions were curated manually and were not necessarily included in the read-depth filter used to filter the SNPs and indels.
(XLSX)

Table S14 **Homozygous Variant Effects.** List of the protein-coding genes for which all known isoforms are affected by a homozygous high-impact SNP, indel or structural variant in at least one quartet member.
(XLSX)

Table S15 **Error Simulation for Sporadic Errors (Absolute Number of Genotypes).** To estimate the number of errors that are detectable by transmission analysis, we simulated all possible genotypes in case of sporadic (1 error in the 8 allele sequenced) or. Averaging the results of the simulation suggests that about 79% of all errors are detected in the children and 69% in the parents (see supplementary methods in Text S1).
(XLSX)

Table S16 **Error Simulation for Non Sporadic Errors.** To estimate the number of errors that are detectable by transmission analysis, we simulated all possible genotypes in case of non-sporadic (more than 1 error in the 8 alleles sequenced) and determined the number of genotypes that contained either an MIE or an SCE.
(XLSX)

Text S1 **Supplementary methods and file description.**
(DOCX)
