# Supplementary figures and images for: Complete Genome Phasing of Family Quartet by Combination of Genetic, Physical and Population-Based Phasing Analysis
Source: PLoS One. 2013 May 31;8(5):e64571. doi: 10.1371/journal.pone.0064571 (PMC3669306; doi:10.1371/journal.pone.0064571)

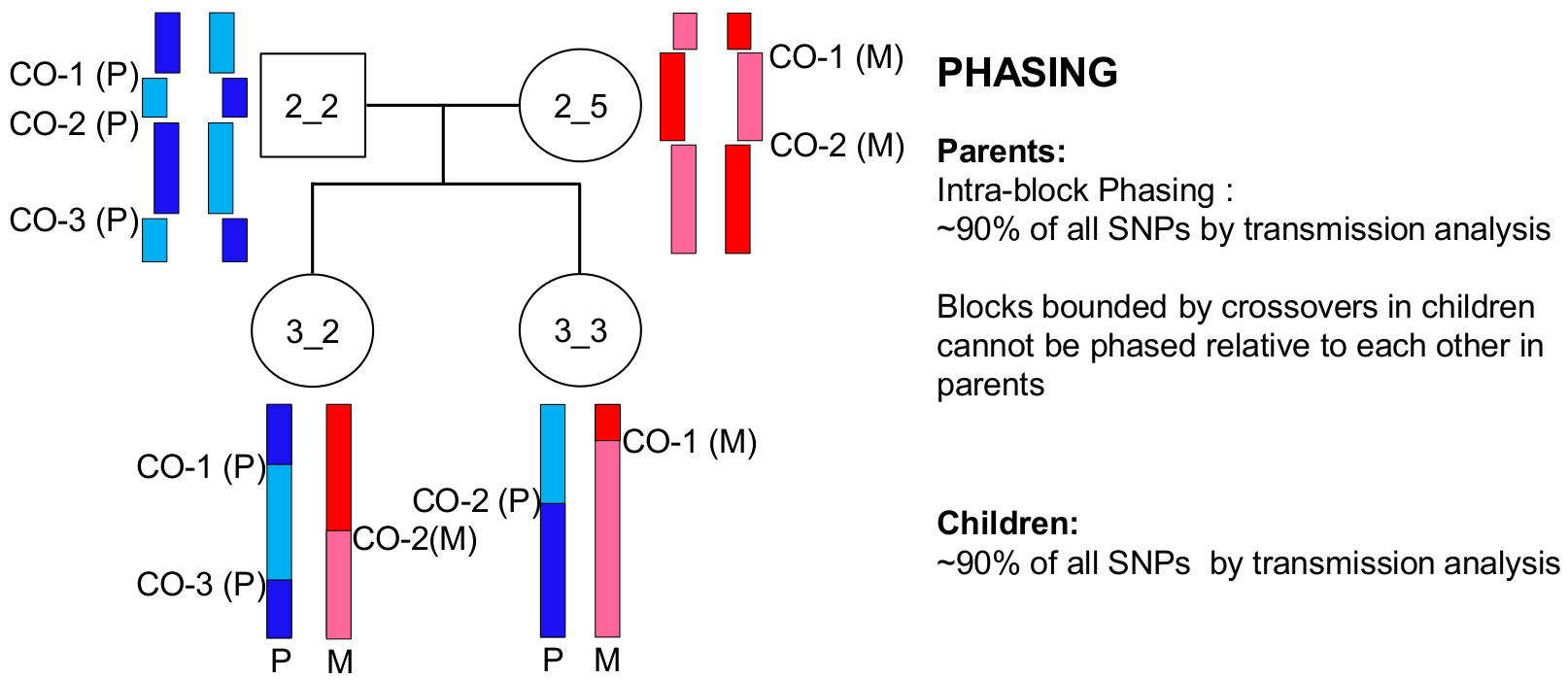

Supplement: Figure S1 — Parents are only partially phased by genetic phasing. Phasing by transmission in a quartet allows complete phasing of the children except for positions that are quadruple heterozygous. In the case of the parents, the variants within blocks bounded by the crossovers in the children are phased but the blocks are not phased relative to each other, therefore, the parent chromosomes cannot be completely reconstituted. Physical method can be used to phase some of the quadruple heterozygous region as well as some of the crossover-defined blocks in the parents. The quartet represented illustrates the relationship of the four member of the healthy Caucasian quartet family FNY01. FNY01_2_2 = Father, FNY01_2_5 = Mother, FNY01_3_2 and FNY01_3_3 = 2 daughters. (TIF) [file pone.0064571.s001.tif]

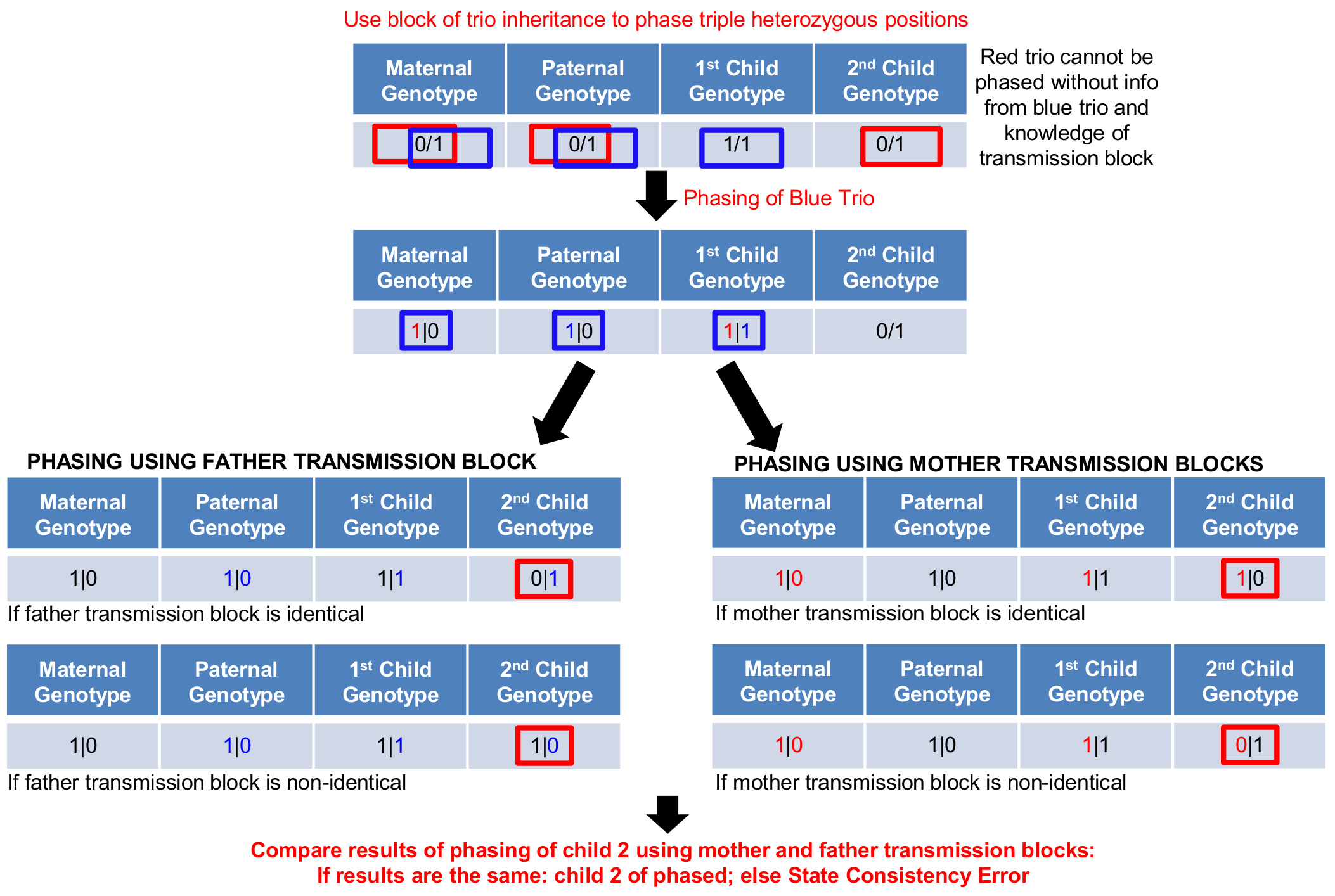

Supplement: Figure S2 — Triple heterozygous phasing. The preliminary inheritance blocks were used to phase the positions in each of the two trios that could not be phased without prior knowledge of the inheritance blocks. After this final phasing step, the edges of the preliminary blocks were refined. (TIF) [file pone.0064571.s002.tif]

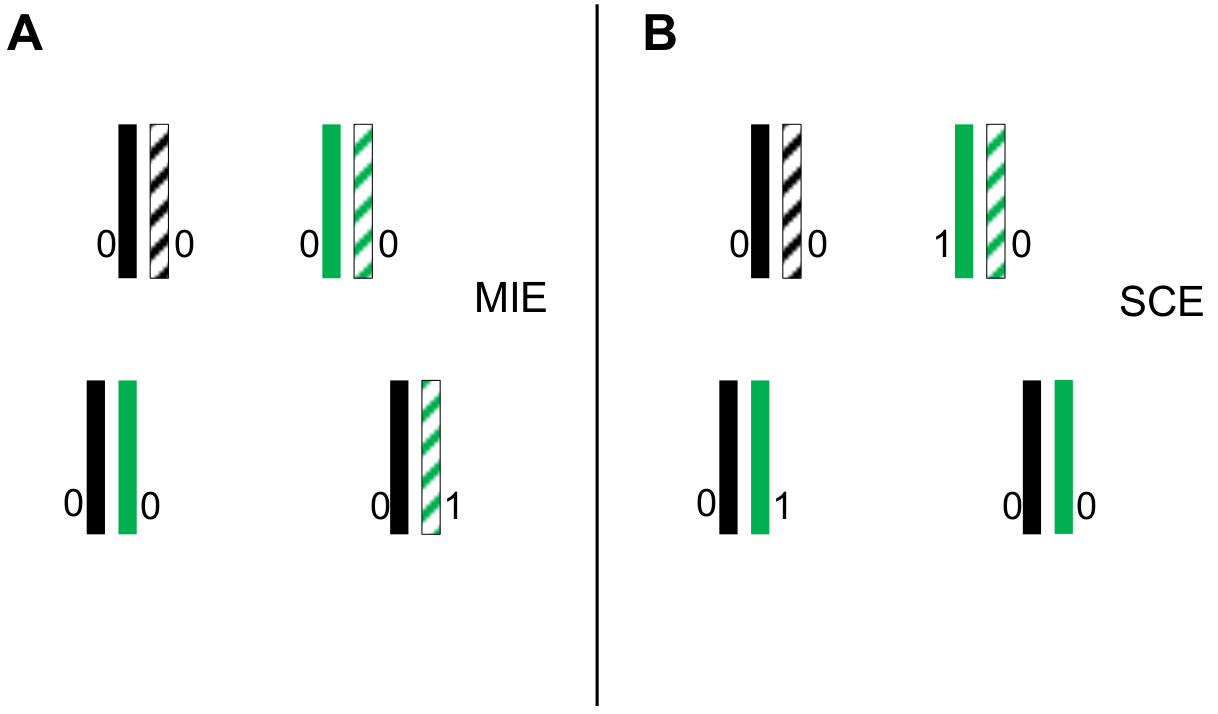

Supplement: Figure S3 — Error analysis. (A) Mendelian Inheritance Error: quartet genotype is incompatible with Mendelian laws. (B) State Consistency Error: quartet genotype is compatible with Mendelian laws but pattern of chromosomal inheritance is not. (TIF) [file pone.0064571.s003.tif]

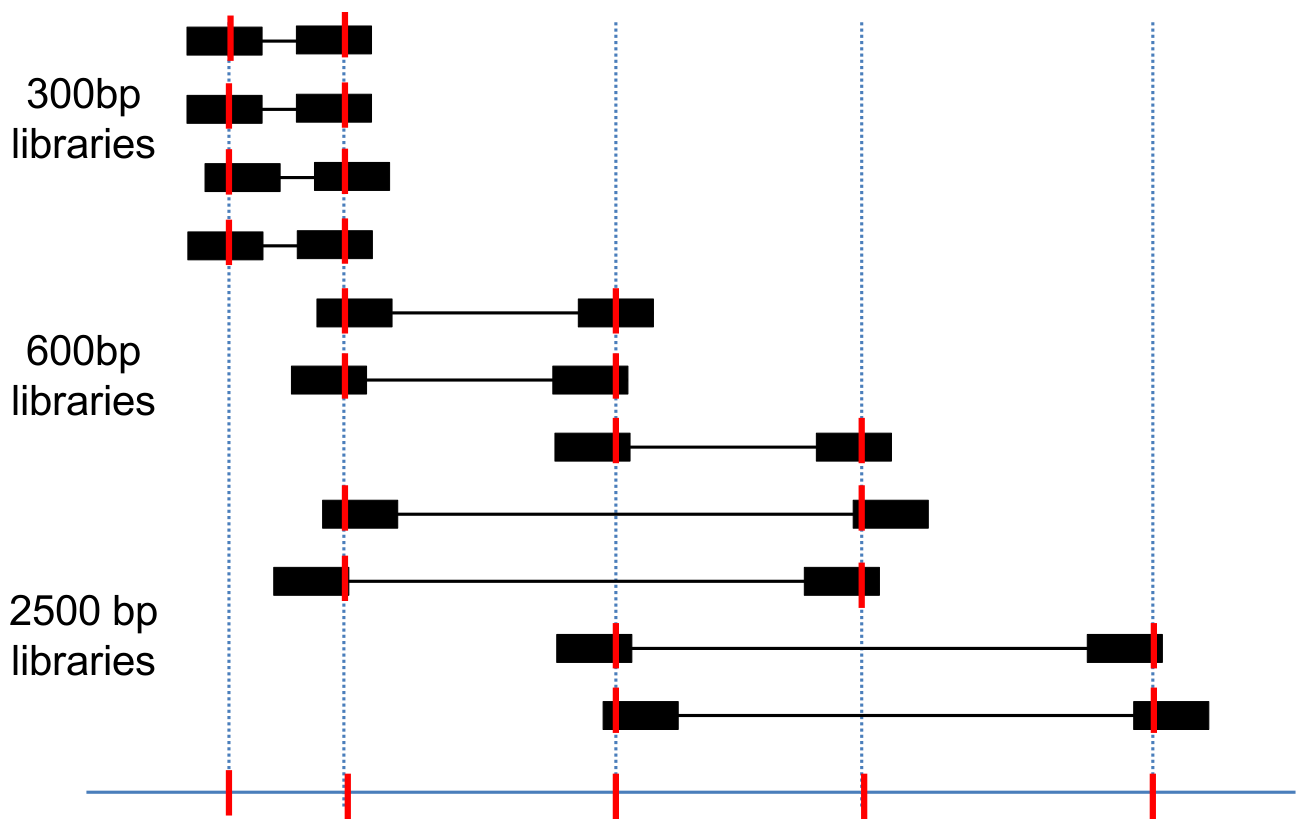

Supplement: Figure S4 — Physical phasing. Heterozygous SNPs found on both ends of overlapping pair-end or mate-pair library fragments can be phased. Read-backed phasing was performed using the GATK package. (TIF) [file pone.0064571.s004.tif]

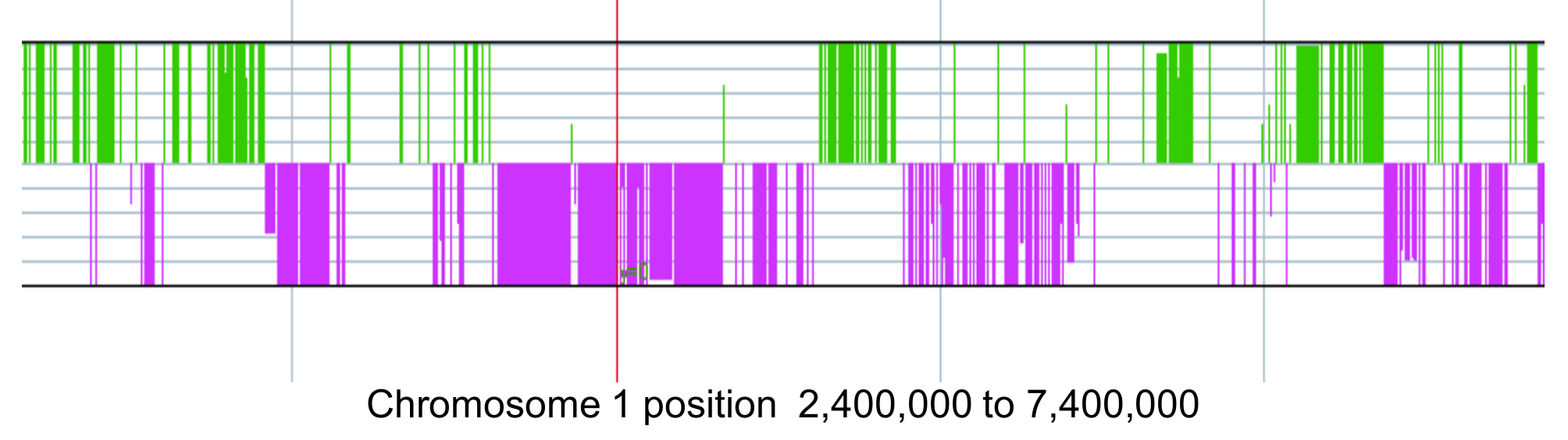

Supplement: Figure S5 — Comparison of HapMap-based and quartet-based phasing. Chromosome 1 of individual FNY01_2_2 was phased using Beagle using the hapmap 3.0 marker file and default parameters and the results were compared with our quartet based phasing. A value of one was assigned to concordant phasing between both methods and a value of negative one for the discordant phasing calls. The switch accuracy defined as the proportion of heterozygous positions misassigned relative to the previous heterozygous position [20] was equal to 3.5%. The first 5 million bases of chromosome 1 are shown. (TIF) [file pone.0064571.s005.tif]

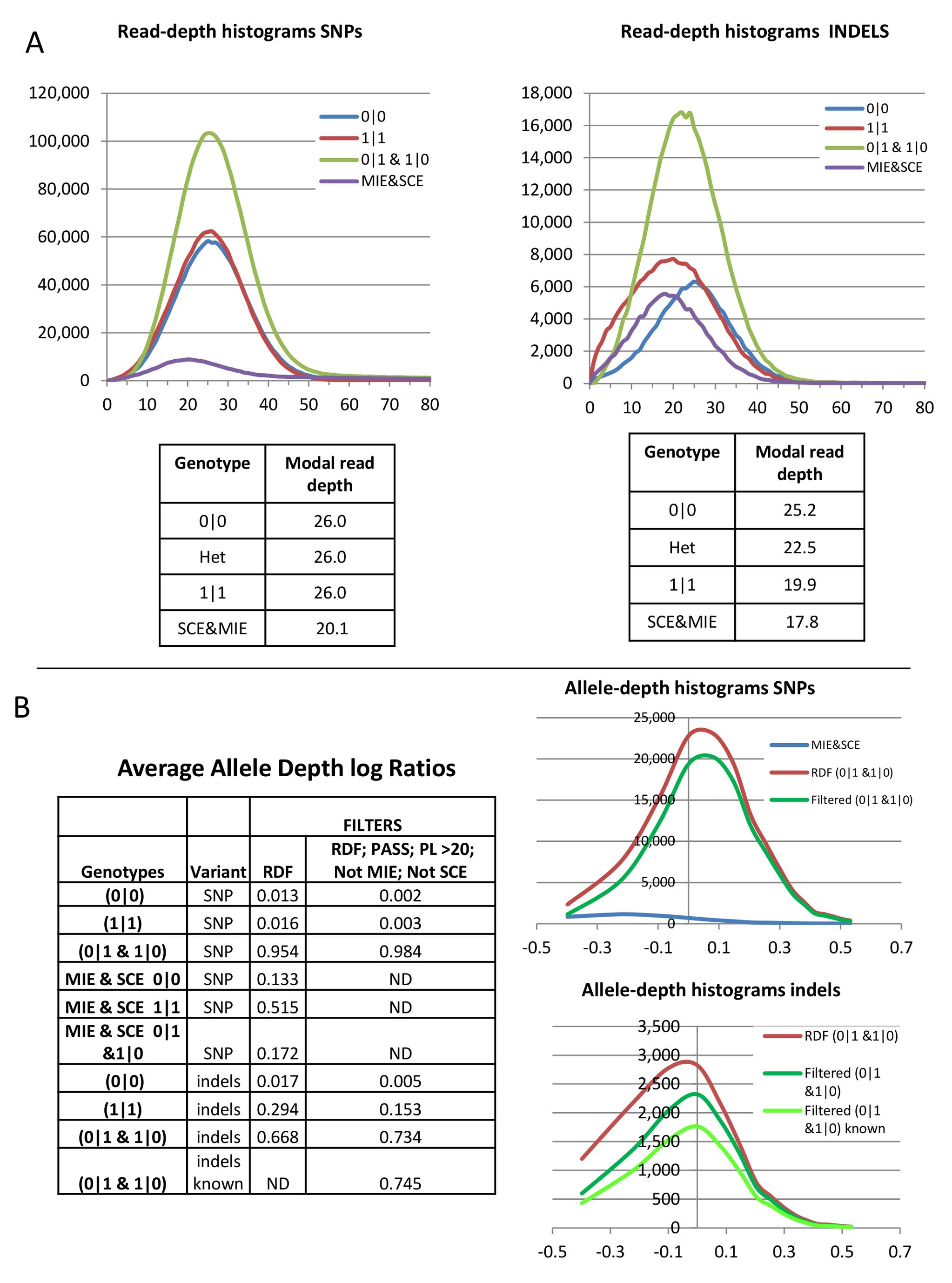

Supplement: Figure S6 — Histograms. (A): Read-depth frequency histograms for 0|0 (REF,REF), 1|1 (ALT, ALT) and heterozygous calls in individual FNY01_2_2 are plotted for SNPs and indels. The 4,8 million SNP list was used after application of the read depth filter. The histograms are almost perfectly super-imposable, suggesting that the alternate (ALT) calls do not occurs preferentially in regions of low read depth. Interestingly the allele depth for the MIEs and SCEs is lower suggesting that the SNP calling errors occur more frequently in regions of low coverage. Hence, higher coverage could decrease the error rate even further. In the case of the indels, the read-depth is in average higher for the reference (REF) calls than for the ALT calls. This confirms that indel calling by the GATK is less precise than SNP calling. (B): Allele-depth frequency histograms. Log ratios of allele-depth are plotted. ALT calls #/REF calls # are plotted in the case of 0|0 and heterozygous genotypes calls. REF calls #/ALT calls # are plotted in the cases of the 1|1 calls. Histograms were generated using either the 4.8 million list of SNPs after application of the read depth filter only, or on the 3.9 million SNP list (RDF, PASS and PL>20 filters). Table on the right summarize average allele-depth ratios. In the case of the SNPs, average allele-depth ratios are very small for the 0|0 and 1|1 calls and close to 1 in the case of the heterozygous calls. As expected, application of more stringent filters yields smaller allele depth ratio in the case of the homozygous calls, and a ratio that is closer to 1 (from 0.93 to 0.98) in the case of the heterozygous calls. We conclude that, as expected, filtering erroneous calls increase the allelic balance quality of the calls. Indel calling is of lower quality than SNP calling. These results also suggest that alignment with BWA followed by variant calling with the GATK yields results with minimal bias in favor of the reference allele. (TIF) [file pone.0064571.s006.tif]

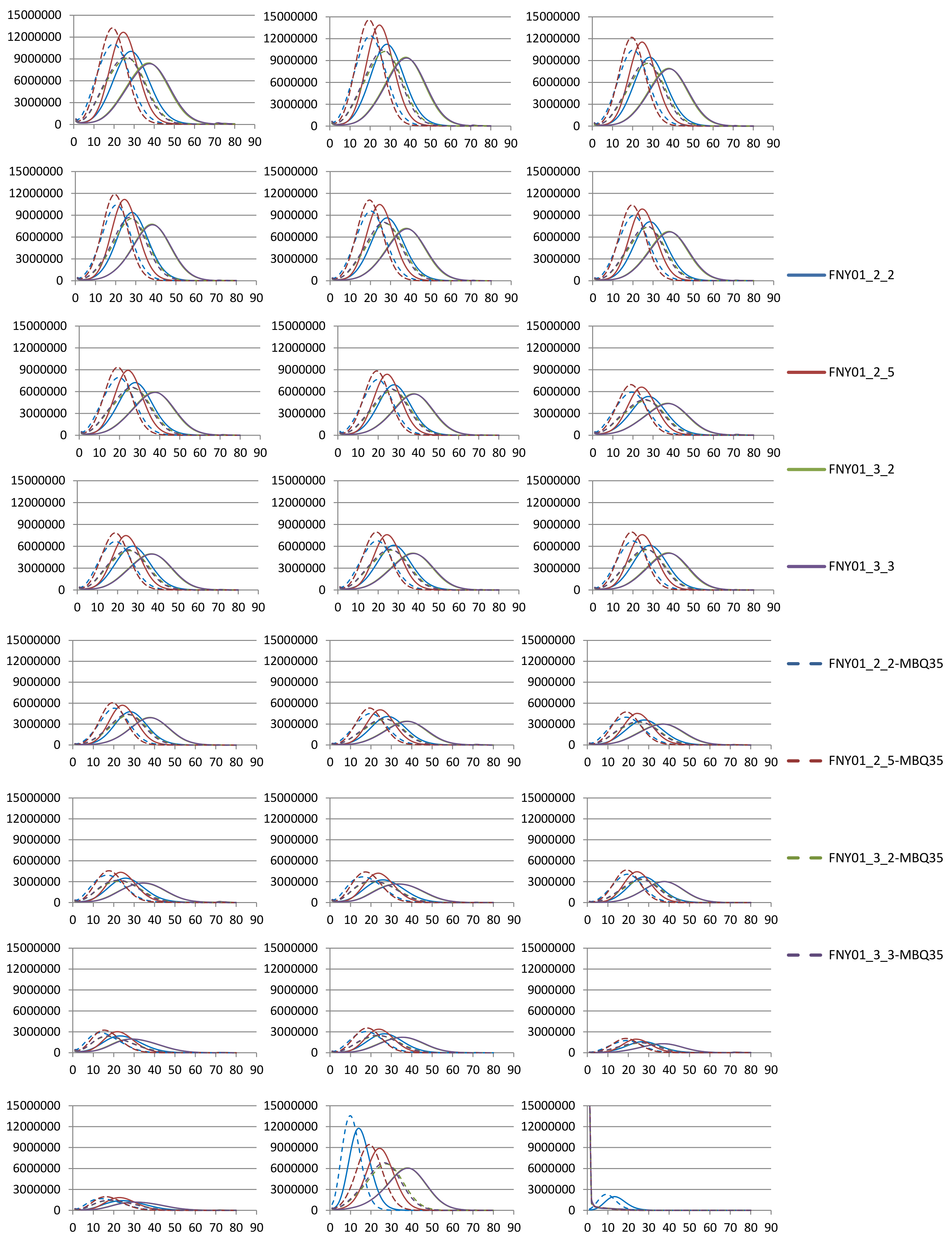

Supplement: Figure S7 — Quality score. Average quality scores and average quality scores greater than 35 for each individual. Average of the results for three libraries (of three different sizes), sequenced once or twice each, and is shown for each individual. X-axis: quality score. Y-axis: number of reads. (TIF) [file pone.0064571.s007.tif]
