# Supplementary material for: Complete Genome Phasing of Family Quartet by Combination of Genetic, Physical and Population-Based Phasing Analysis
Source: PLoS One. 2013 May 31;8(5):e64571. doi: 10.1371/journal.pone.0064571 (PMC3669306; doi:10.1371/journal.pone.0064571)
Supplement: Text S1 — Supplementary methods and file description. (DOCX) [file pone.0064571.s024.docx]

# Supplementary methods

## Error rate analysis

Error in sequencing can be defined as false positive calls (variants present in the VCF files but not in the actual sample), false negative calls (variants that exist in the sample but not in the VCF files) and no-calls (regions where the variant could not be called because of low read-depth. Table S3 lists the number of no-calls and partial calls.

Sequencing errors can be introduced at all steps of the sequencing and analysis pipeline including library preparation, sequencing reaction, base calling , alignment to the reference sequence, variant calling and genotyping. The majority of errors are caused by PCR errors, by sampling fluctuations due to the lack of coverage in specific regions, or by DNA structure that contains regions which are difficult to sequence (simple repeats or copy number variation). PCR errors are expected to cause sporadic errors defined as errors in only 1 of the 8 sequenced alleles. The other types of errors are expected to cause non-sporadic errors defined as errors that frequently occur in more than one of the 8 alleles sequenced.

## What proportion of all errors can be detected by transmission analysis?

To answer this question, which is critical to calculate the residual error rate after transmission analysis, it is useful to first know what proportion of errors is sporadic and what proportion is non-sporadic. The proportion of sporadic versus non-sporadic errors can be estimated by measuring the number of errors that occur in the part of the genome that does not contain any SNPs because in these regions, sporadic errors will result in a genotype call where 7 alleles are similar and one is different. Since this invariant fraction of the genome represents more than 98% of the genome, it should contain most of the stochastic errors. We found that about 24.6% of the errors detected by transmission (GDEs) occurred in quartet genotypes compatible with a stochastic error in an invariant part of the genome. Therefore at least 75.4% of errors are not sporadic.

In the case of sporadic errors, the proportion of GDEs was calculated by simulating all possible errors as follows (Table S15). We first computed the 81 possible quartet genotypes (considering that each individual could have three genotypes (0/0; 0/1 and 1/1) and tested their compatibility with Mendelian laws. We found that 29 of the 81 possible quartets are Mendelian. Then, we computed whether each of these quartet genotypes was compatible with each of the four states of inheritance (identical; non identical, paternal identical and maternal identical). Of the 116 (4 x 29) possibilities, 62 did not show any SCE. A sporadic error was then introduced into each of the 8 allelic positions in these 62 possibilities and the proportion of SCEs, MIEs and undetectable errors was computed. The results show that all sporadic errors are detectable in the children, and that 38.7% of all errors are undetectable in the parents. Another important result from this table is that all errors in both parents and children are GDEs in the non-identical blocks.

To estimate the number of GDEs for non-sporadic errors (which represent 75% of all errors), we used two methods. The first method was simply to calculate the ratio of the number of genotypes that are neither SCE nor MIE to all possible genotypes in each state of inheritance (4 x 81). As discussed above there are 62 such genotypes. This ratio is therefore equal to 62 / 324 = 28%.

The second method was to introduce one to eight random errors in each of the possible 116 combinations of inheritance states and Mendelian quartet genotypes, and to calculate the number of detectable and non-detectable errors. The results in Table S16 show that on average 30.07% of all errors are not GDEs.

Assuming that 75% of all errors are sporadic and 25% non-sporadic, it follows that the number of undetectable errors is about 21% in the children (75% of 30.07%) and about 32% in the parents (75% of 30.07% plus 25% of 38.7%). For simplicity we averaged and rounded these numbers to 25% and calculated error rates for the entire quartet.

## Error rate estimation using invariant positions

Less than 1% of the genome contains SNPs. Therefore more than 99% of the genome is invariant in any particular individual. Assuming that sequencing errors happen randomly in the genome, the vast majority of errors should fall in the invariant part and therefore lead to genotypes where 7 of the 8 alleles sequenced are identical and one is different. Such regions, are particularly useful to estimate the error rate because errors in one of the children will always result in an MIE, while errors in the parents will results in a SCE in 50% of the cases (whenever the erroneously called allele was on a block that was transmitted to one of the two children). Therefore the number of MIEs in invariant regions should be twice the number of SCEs. The number of undetected errors in invariant regions can easily be calculated from this data since it should be equal to the number of SCEs.

## Error rate analysis in regions which are identical by descent

These regions are particularly useful to estimate sequencing error rates, since the diploid sequence in these regions was sequenced in duplicates (once in each children). In these regions the number of MIEs should theoretically be equal to the number of SCEs. Rate of errors can be calculated by comparing these regions in the two children. About 33.4% of the genomes of the children were identical by descent (i.e. had inherited the same chromosomes from both parents)

## Error rate analysis in regions which are non-identical

These regions are interesting because all errors are GDEs in both parents and children (see above). Error rate in these regions can be calculated without having to use any correction factor for undetectable errors (assuming that the number of multiple errors is very rare). About 18.3% of the genome of the two children in the quartet is in the non-identical state.

# File Description

## Data Access

The raw sequence files, the 2 vcf files and the bed file with the family inheritance state blocks are available at NCBI BioProject database (<http://www.ncbi.nlm.nih.gov/bioproject/191008>) with Accession number: PRJNA191008 and ID: 191008 or at <http://genplay.einstein.yu.edu/FNY01/>

## FNY01_SNP_INDEL_2013-02-26.vcf

## FNY01_SV-2013-02-26.vcf

These files follow the VCF 4.1 specification. They contain respectively the result of the SNP and indel calling and the result of the structural variant calling.

**The following custom error flags were added after the transmission analysis:**

RDF (Read Depth Filter): variant is in a high depth region

MIE: variant is a Mendelian Inheritance Error

SCE: variant is a State Consistency Error

PLF (PL filter): variant did not pass the PL > 20 filter

HEF (Hemizygous Filter): variant was called as heterozygous in a hemizygous region

**The following flags were added after the Beagle analysis:**

Imputed: call was imputed by Beagle

Uncalled: means that the position was uncalled before imputation (only present when Imputed is present)

Unphased: position was unphased before imputation (only present when Imputed is present)

BGL_PR: Beagle Process means the SNP was in the HapMap or 1000 genome project and was processed by Beagles (about 80% of all SNPS were in that category)

MIE_corrected means that the SNP was an MIE before Beagle (only present when Imputed is present)

SCE_corrected means that the SNP was an SCE before Beagle (only present when Imputed is present)

**We also added two info fields to the VCF file:**

 Phase set: The PS field indicate the phase set. When the phase set is the same it means that the SNPs were in the same phased transmission block. The identifier is the first position of the block (the PS only matters for the parents because the children are always phased (in a quartet).

 Ancestral allele: The AA field indicate the parent of origin. For the parents we randomly assigned P1/P2 and M1/M2 names. In the kids we assigned the P1 and P2 and M1 and M2 tags according to the phasing and the crossover map generated by the phasing.

We recommend loading these files as a multi-genome project using the software GenPlay (available at genplay.einstein.yu.edu).

## Quartet_Inheritance_State_Blocks.bed

This bed file contains the quartet inheritance state blocks as described in the main text of this article. The inheritance state blocks are defined as follow: maternal inheritance state | paternal inheritance state. We recommend loading this file as a Repeat Layer using the software GenPlay (available at genplay.einstein.yu.edu).
